# The electrical tumor microenvironment: abnormalities and opportunities

**DOI:** 10.3389/fonc.2026.1870705

**Published:** 2026-08-26

**Authors:** Alice Burchett Darantiere, Fionn Lay, Meenal Datta

**Affiliations:** 1 Department of Aerospace and Mechanical Engineering, University of Notre Dame, Notre Dame, IN, United States; 2 Department of Chemical Engineering, University of Notre Dame, Notre Dame, IN, United States

**Keywords:** cancer, capacitance, electroceuticals, endogenous and external electric fields, immune cells, immunotherapy, impedance, membrane potential

## Abstract

Abnormal electrical properties in cancerous tumors have been observed for more than a century. Modern technological advances have enabled rapid improvements in our understanding of these electrical facets and our ability to leverage this information for diagnosis and treatment. Here, we outline the current knowledge of the electrical tumor microenvironment (eTME), including electrical abnormalities at the tissue, cell, and molecular scales. Of particular interest to solid tumors and immunotherapy, the innate immune system responds profoundly to these abnormalities with altered mobility and effector function. Improved understanding of the eTME has enabled new advances in disease detection, with techniques such as impedance spectroscopy showing promise as minimally invasive, sensitive, and specific methods that complement traditional approaches. Therapeutic strategies that use electrical stimulation via externally applied fields, electrically active nanomaterials and devices, and so-called electroceuticals have shown promise in augmenting cancer therapy efficacy, particularly via leveraging the immune system. This review summarizes the state of the field, highlighting the eTME as a foundational – yet often overlooked – feature of cancer and a rapidly-growing area of novel therapeutic development.

## Introduction

More than two centuries ago, observations by Galvani and Volta shed light on the existence of electrical activity in living muscle (1). Biologists today are familiar with excitable cells and the role that electrical signals play in neurological and muscular function, but the electrical properties of all cells (including non-excitable) and tissues are critical for physiological processes such as development and wound healing. H.S. Burr, a pioneer in the field of bioelectricity, demonstrated that electric fields are dynamically involved in embryonic development, wound healing, and cancer (2–5) Wounding generates an endogenous electric field that serves to direct the migration of stromal and immune cells (6–9) Cancer has been often described as a wound that does not heal, and tumors indeed exhibit altered electrical characteristics that can be likened to those created by a wound (10). The tumor microenvironment (TME) is already known to be abnormal biologically (e.g., aberrant vasculature, lymphatics, extracellular matrix, and stromal and immune cell interactions), chemically (e.g., hypoxia, acidosis, altered metabolism), and mechanically (e.g., pathophysiological stiffness, viscoelasticity, solid and fluid forces). However, the TME is also electrically abnormal, which has been largely overlooked in fundamental cancer research. These electrical abnormalities give rise to unique opportunities for sensitive and non-invasive diagnostic techniques, enhanced electrical-based therapies, and bioelectric-targeted pharmacological interventions.

All tissues have passive electrical properties (11). Biological fluids, which comprise most of the body and surround and fill each cell, carry electrolytes (dissociated and dissolved ions) and permit the flow of electrical current (12). Cell membranes on their own have very low conductivity (they resist the flow of current), but ion channels embedded in the cell membrane allow for the tight control of ion transport, rendering each cell a distinct electrical unit with resistive and capacitive properties. The abundance of charged molecules inside and outside of the cell, the active and passive transport of these ionic species, and the organization of cells into tissues collectively give rise to measurable electrical characteristics. These include the ability to conduct or oppose electrical current (conductivity and resistivity, respectively), the frequency-dependent opposition to alternating current (impedance), the capacity to store electrical charge (capacitance), and the tendency to distort an applied electric field (permittivity).

**Figure 1:**
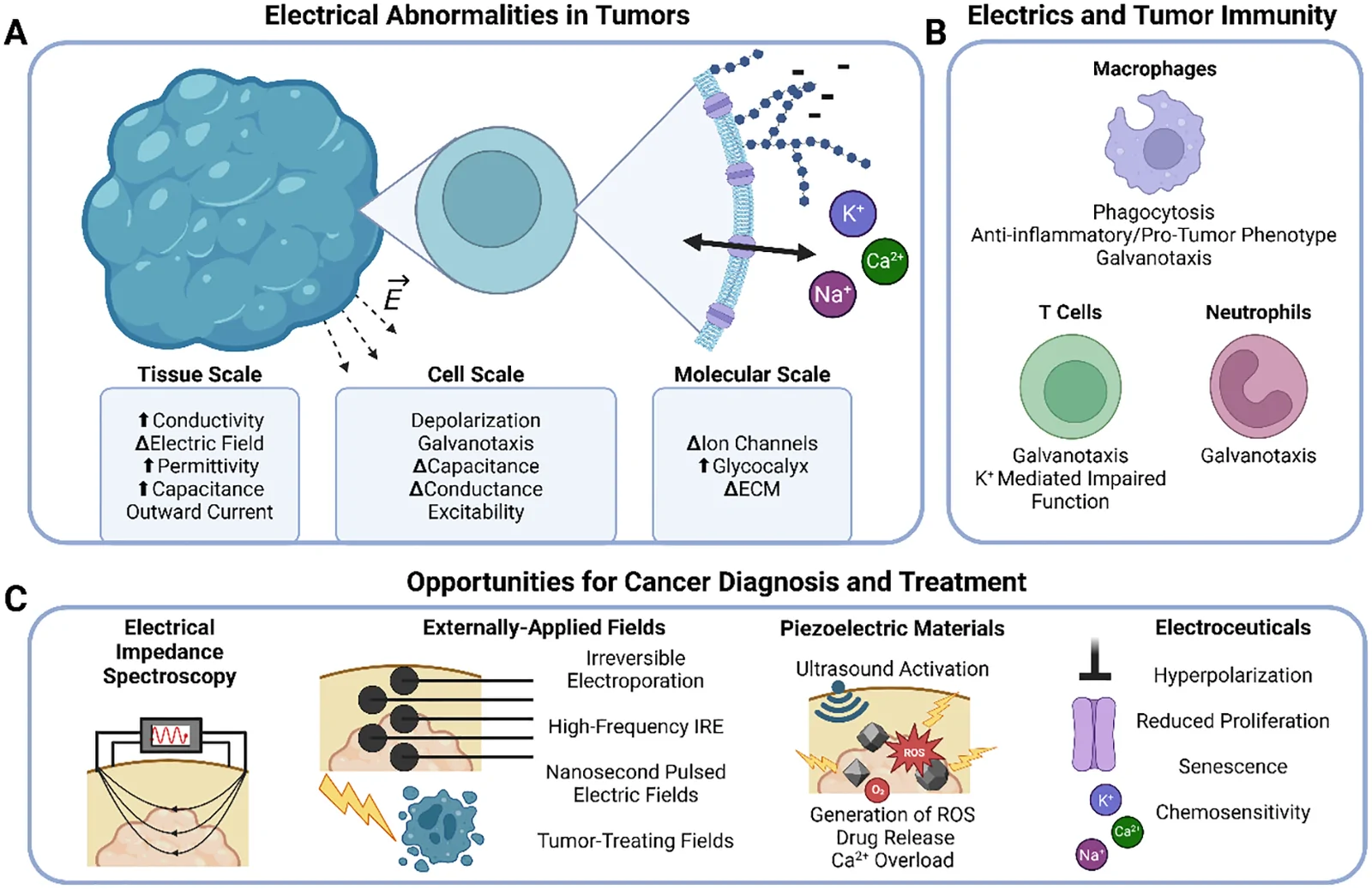
Overview of the electrical abnormalities of cancer and opportunities to target them. **(A)** Cancer is characterized by electrical abnormalities on the tissue, cellular, and molecular scales within the tumor microenvironment, with effects on various cells including immune cells **(B)**. **(C)** These abnormalities also present opportunities for novel and combinatorial diagnosis and treatment strategies based on electricity.

Cells actively maintain a finely-tuned gradient in charged ions such as sodium, potassium, calcium, and chloride across the cell membrane separating the cytoplasm from extracellular space (13). Despite its name, resting membrane potential is neither passive (energy is required for its active maintenance) nor static (membrane potential changes during a range of physiological and pathological processes) (14). Blood plasma and interstitial fluid has several orders of magnitude more sodium, chloride, and calcium than intracellular fluid, while potassium has a much higher concentration inside the cell (15). These concentration gradients are maintained through the activity of ion pumps and enable rapid response of a cell if its ion channels open to allow ions to flow down their electrochemical gradient. Voltage differences and the resulting ionic current and electrical fields in cells and tissues can thus be considered as “active” electrical traits, as compared to the passive electrical properties listed above. This review summarizes current research on the electrical tumor microenvironment (eTME) and related phenomena and highlights recent discoveries in diagnosis and treatment leveraging bioelectricity abnormalities in cancer, summarized in **Figure 1**.

## The electrical tumor microenvironment

### Electrical abnormalities at the tissue scale

Given the myriad of biological, chemical, and mechanical differences that distinguish tumors from healthy tissues, it is no surprise that they also display altered electrical characteristics. This has been an area of scientific inquiry for more than a century. An early study in 1922 by Crile et al. observed increased conductivity of cancerous tissue compared to normal adjacent tissue, with the tumor periphery featuring higher conductivity than the tumor core (16). In 1936, Burr et al. found that mice exhibit drastic increases in tissue voltage at the site of the primary tumor up to two weeks before a palpable tumor can be detected (17). Burr and colleagues went on to show distinctions in electrical patterning between cancer-susceptible and cancer-resistant mice, as well as a reversal of tissue-level electrical fields in women with and without cervical cancer (5, 18).

As tumors grow, they can physically disrupt structures that normally maintain physiologic electrical gradients. For example, breast tumors damage or destroy epithelial barriers in breast tissue, creating alterations in the endogenous electrical field that is detectable from the surface of the skin (19, 20). Proliferative tumor tissue is depolarized compared to the surrounding non-proliferative tissue, which results in local electrical fields within and around tumors (21). Tumors are generally measured to have outward current, though some studies report the reverse (21–23). This parallels the currents measured in wounds, which are also generally measured to be outwards from the wound, and at a similar magnitude (6–9). This endogenous field impacts tumor invasion and immune recruitment and activity, as described below.

Tissue-level differences in dielectric properties of tissue can be measured using coaxial probes or by dielectric spectroscopy (24). Electrical impedance, or total resistance to alternating electric fields, can be measured directly through probes in contact with tissue. As the technology continues to advance, more sensitive measurements of different electrical properties can distinguish between normal and cancer tissue and also predict tumor grade or malignancy. For example, the relative permittivity and effective conductivity of breast tumors are significantly higher than those of benign tumors, which are in turn higher than those of normal breast tissue (25). In a study that included post-mortem samples of various brain tumor types and healthy brain tissue, the electrical impedance of human glioma tissue was found to be significantly lower than that of normal brain tissue (26). The authors could also distinguish between grades of tumor, as the impedance of high-grade glioblastoma was significantly higher than that of lower-grade astrocytoma. The increased capacitance of tumors can also be used to distinguish between normal and cancerous tissue, with imaging techniques that can identify small lesions with greater specificity than positron emission tomography (PET) imaging (27). The electrical characteristics of various cancer tissues and their diagnostic potential are reviewed elsewhere (28–30).

### Electrical abnormalities at the cellular scale

To better understand these tissue-level abnormalities in tumors, we must explore their underlying causes at the cellular level. Cells generally maintain a negative resting potential, meaning that the interior of a cell is negatively charged relative to the extracellular space – referred to as membrane polarization. This is essential for normal cell function. As reviewed by Yang and Brackenbury, cancer cells feature a depolarized membrane compared to normal cells, regardless of cancer type (31). While the normal membrane potential of differentiated cells is approximately -40 mV to -100 mV, most cancer cells have a membrane potential between 0 mV and -40 mV (32). This is the same range that is measured for proliferating and stem-like cell populations, rendering membrane depolarization one manner in which cancer cells resemble stem or embryonic cells. Depolarization is one putative mechanism of proliferation, as artificial depolarization of mature cells is sufficient to induce DNA synthesis and initiate mitosis (33, 34). Depolarization also renders cells more susceptible to oncogene-induced tumorigenesis and can even induce cancer-like phenotypes in adjacent cells (35). Conversely, membrane polarization in normal, quiescent cells may serve to inhibit proliferation by preventing the transition from the G1 phase of the cell cycle to the S phase and DNA synthesis (36). However, because shifts in membrane potential are mediated by changes in the concentrations of ions that have both electrochemical and biochemical properties (e.g., inducing signal transduction), elucidating the mechanism behind the relationship between membrane potential and cell behavior is non-trivial. The role of disrupted ion channel abundance and activity and the resulting changes in membrane polarization in different stages of cancer is well-reviewed elsewhere (15, 31, 37, 38).

Changes in membrane potential arise concurrently with oncogene activation in tumorigenesis. Levin and colleagues, pioneers in modern bioelectricity insights and models, have shown in planaria that tissues with oncogenic KRAS mutations can be made to exhibit completely normal histology/anatomy if forced to the normal bioelectric state of neighboring tissues (39). When *Xenopus laevis* are injected with human oncogenes, depolarization occurs as an early step in tumorigenesis in a wide range of cells. Targeting the expression of hyperpolarizing channels and oncogenes significantly suppresses the formation of tumor-like structures in *Xenopus*. These findings – particularly for KRAS, a heavily conserved oncogene that also promotes human tumorigenesis – highlights a promising area for therapeutic intervention, as discussed later.

Electric fields in tumor tissue give rise to galvanotaxis, the directional movement of cells in response to endogenous electric fields (40). Metastatic tumor cells are particularly susceptible to galvanotaxis, as seen with prostate cancer cells, breast cancer cells, lung cancer cells, and glioblastoma (41–44). The manner of galvanotaxis, whether towards an anode (anodal) or a cathode (cathodal), appears to be heavily microenvironment and cell-dependent (40). Typically examined with microfluidic devices, both anodal and cathodal migration have been observed for different normal and cancerous human cell types. Human granulocytes, corneal endothelial cells, and vascular endothelial cells display anodal galvanotaxis, while activated T lymphocytes and keratinocytes display cathodal migration (45–49),. However, directional migration of cells can be reversed by altering the microenvironmental conditions. For example, brain tumor-initiating cells display anodal migration when cultured on a poly-L-ornithine/laminin-coated surface, but display cathodal migration when embedded in a 3D extracellular matrix made up of hyaluronic acid and collagen (44, 50). Thus, inhibiting galvanotaxis to reduce cancer invasion may require a cancer-specific or even patient-specific approach. These results also implicate the importance of 3D vs 2D *in vitro* systems to study the eTME, mimicking findings from the study of other tumor abnormalities.

In addition to resting membrane potential and response to electric fields, other electrical properties also distinguish cancer cells from normal cells. Membrane capacitance is a measure of the charge required to depolarize the cell membrane. Cancer cells have been measured to have both higher and lower membrane capacitance than normal cells (51, 52). Capacitance may increase with metastatic phenotype, drug resistance, and malignancy (53–55). However, decreased cancer cell capacitance is correlated with reduced overall survival in patients with head and neck cancer (56). Cytoplasmic conductance, the ability of the cytoplasm to conduct current, is another electrical biomarker. Studies report that cytoplasmic conductance increases with malignancy but decreases with metastatic phenotype (53, 55). Cytoplasmic conductance changes with chemotherapy resistance, but whether it increases or decreases depends on the specific drug and mechanism of resistance (54, 57). For whole-cell measurements, decreased impedance indicates cancerous and metastatic phenotypes, and increased conductivity is associated with increased malignancy (55, 58–60).

Dynamic electrical states of cancer cells, with changes able to be measured on the millisecond scale, are also emerging as a critical part of tumor progression, particularly in the context of brain malignancies. Growth of high-grade gliomas is dependent on electrochemical synapses between tumor cells and nearby neurons, resulting in potassium currents that propagate through electrically coupled tumor cells (61). Glioma cell membrane depolarization resulting from this electrical activity promotes proliferation and tumor growth, with greater depolarization correlating to increased proliferation levels (61, 62). Subpopulations of electrically active glioma cells (exhibiting neuron-like excitability) display autonomous, rhythmic calcium oscillations, acting as hubs for other interconnected glioma cells and contributing to their survival (63). In humans, glioma infiltration causes an increase in brain excitability, such that there is an electrically-enabled positive feedback loop resulting in tumor progression (61). This effect is not exclusive to brain cancers, however, as small cell lung cancer cells also have depolarizing currents in response to neuronal activity (64). The electrical excitability of cancer cells has been proposed as a driving mechanism and metric of malignant behavior and is linked to invasion and metastasis in a range of carcinomas (65). It is now recognized that many solid tumors are innervated – often more densely than the surrounding normal host tissue – and that this innervation may be a critical driver of tumor progression, representing a new field of cancer neuroscience beyond the brain (66–69).

The observations described above are made possible by highly precise measurement modalities. The patch clamp technique is one conventional method to measure the electrical properties of single cells (36). A glass pipette containing an electrode uses suction to create a seal with a portion of the cell membrane, permitting measurement of membrane potential or response to applied current. Alternatively, a sharp microelectrode can be placed inside the cell to obtain the potential difference compared with an extracellular reference electrode. Nanoprobes can also be used to measure the flow of current on a subcellular scale (70). While these methods obtain very precise measurements, they do not readily provide spatial, longitudinal, or multi-cell measurements. Voltage-sensitive fluorescent dyes are therefore useful tools to non-invasively measure electrical activity in cells with high spatial and temporal resolution. These are typically lipophilic molecules that are embedded in the cell membrane, where they interact with charge on the interior and exterior of the cell and have a voltage-dependent fluorescence spectral shift when the membrane potential changes (71). Genetically engineered voltage indicators can be used in a more targeted manner to mechanistically determine the activity of specific ion channels, and there are ongoing efforts in the field to develop targeted, non-genetically encoded voltage indicators (71) (72).

### Electrical abnormalities at the molecular scale

The abnormalities in cell electrical properties described above arise due to alterations on the molecular scale, both within and outside of the cell. Because the lipid bilayer of the cell membrane is effectively impermeable to charged species, the passage of ions (and therefore the conductivity) of the cell membrane is controlled by transmembrane proteins known as ion channels (73). Alterations in abundance and function of ion channels are proposed to underlie major hallmarks of cancer (proliferation, resistance to apoptosis, invasion, and angiogenesis) (15). For example, the activity of voltage-gated sodium channels may enable the electrical excitability described above (33, 65) in addition to their known involvement in increased proliferation, migration, invasion, and metastasis (41, 42, 74, 75). The alterations in expression of ion channels and their associated molecular mechanisms in cancer have been reviewed extensively by others (33, 76).

Mechanosensitive ion channels are of particular interest as they link mechanical properties to electrical phenomena. As described by Momon et al., these channels sense mechanical cues such as microenvironmental stiffness, fluid shear stress, and solid stress to regulate the permeation of ions and control intracellular biochemical and electric signaling (77). For example, the Piezo family of mechanosensitive ion channels change conformation in response to changes in membrane curvature or tension, allowing the passage of positive calcium ions into the cell (78). This results in a measurable increase in intracellular calcium (79), triggering a range of downstream effects, such as mitochondrial depolarization (80), leading to an increase in oxidative phosphorylation (81). In cancer, Piezo1 has been shown to promote proliferation and migration in gastric cancer cell lines (82, 83). High Piezo1 expression has also been linked with low survival in patients with breast cancer (84). Its role in the progression of numerous cancer types and its association with poor patient survival renders Piezo1 an appealing, but challenging, therapeutic target (78).

Structures just outside of the cell also contribute to electrical abnormalities. In cancer cells, the increase in positively charged ions inside the cell is often accompanied by a concurrent increase in the negatively charged glycocalyx on the cell exterior, as reviewed elsewhere (85, 86). This further contributes to cell depolarization as the cell interior becomes less negatively charged than the exterior. Overexpression of negatively charged glycocalyx molecules can facilitate immune evasion, shrouding “eat me” signals on the cell surface and causing electrostatic repulsion between a target cancer cell and phagocytic immune cells (87). Removal of the glycocalyx of cancer cells facilitates their clearance by macrophages (87).

The extracellular matrix, deposited by cancer cells or tumor-resident stromal cells, also has important electrical implications. The extracellular matrix is a network of proteins coated with negatively charged polysaccharides, allowing it to sequester water and positively charged ions (88). Solid tumors feature drastic remodeling of the extracellular matrix, as reviewed elsewhere (89, 90). The transmission of electrical signals between cells closely depends on the surrounding extracellular matrix (91). Even small changes in collagen concentration cause a significant change in the conductivity and dielectric permittivity of a solution (92). The extracellular matrix also serves as a selective filter, inhibiting the movement of charged molecules, while uncharged molecules pass through it more easily, with implications for drug delivery in the tumor parenchyma (93). More research is needed to elucidate the interactions between cancer-associated extracellular matrix changes and electrical properties on the cellular and tissue scale.

## Electrics and tumor immunity

**Figure 2:**
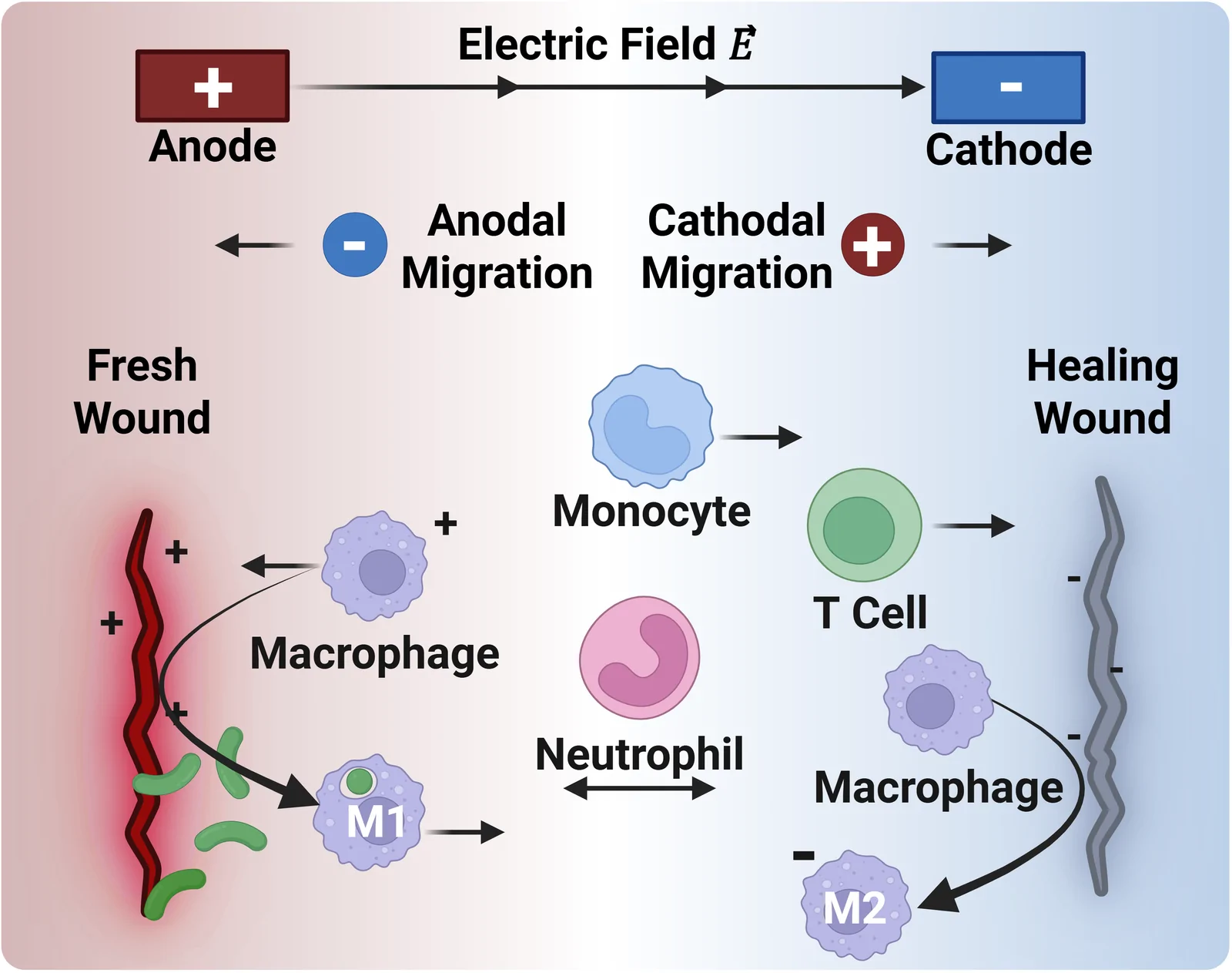
Overview of immune cell galvanotaxis in electric fields, such as those generated by a fresh wound, a healing wound, and a tumor. Macrophages migrate anodally towards fresh wounds, and cathodally away from the wound after phagocytosis of bacteria. When near a negatively charged healing wound, macrophages repolarize to an M2-like phenotype. Monocytes and T cells migrate cathodally, and neutrophils may migrate in either direction.

The electrical characteristics of the TME described above influence not only cancer cells, but also the immune system **Figure 2**. While the interaction between immune cells and their electrical environment has been studied for decades, many questions remain as to the role of bioelectricity in tumor immunity and immunotherapy. Innate immune cells such as macrophages, neutrophils, and dendritic cells make up a significant portion of most solid tumors and play a pivotal role in tumor initiation, progression, and response to treatment (94). These cells modulate the activity of adaptive immune cells such as T cells, and together they are major determinants of patient outcomes. In other contexts besides cancer, myeloid cells rapidly respond to sites of injury and are the first line of defense against pathogens. Their response is impacted by tissue polarization, as shown in a zebrafish model of injury and infection (95). Organism-wide depolarization of membrane potential increased myeloid cell mobilization and improved resistance to infection (95). Depolarized cells at the site of an injury also caused mobilization of myeloid cells and increased infection resistance (95). As discussed above, cancer cells are also depolarized, a feature that may contribute to myeloid cell accumulation in tumors.

Macrophages are one of the most abundant immune populations across solid tumors and are often (but not always) associated with poor prognosis (96, 97). They can adopt a pro-inflammatory phenotype associated with inflammation and anti-tumor immunity, or an anti-inflammatory phenotype associated with wound healing, immunosuppression, and tumor promotion, as well as hybrid states in between the two ends of this continuum. Here, we use the term “phenotype” rather than the commonly used macrophage “polarization” to avoid confusion with the electrical polarization, such as membrane polarization. When in a polarized, negatively charged environment such as a healing wound, they adopt an anti-inflammatory, M2-like phenotype to promote healing (98). This contrasts with the infection-resistant pro-inflammatory response triggered by a fresh, depolarized wound in the zebrafish model above (95). However, an electric field alone will not determine phenotype, though it may augment pro- or anti-inflammatory responses to biochemical cues (99, 100). When placed in an electric field *in vitro*, macrophages generally migrate anodally, towards the positive charge (99). However, the directional migration of macrophages also depends on context; they will migrate anodally towards a site of infection, and then migrate cathodally after having phagocytosed bacteria (101).

The membrane potential of macrophages both reflects and influences phenotype. Inflammatory macrophages are depolarized, while anti-inflammatory macrophages are hyperpolarized (102). When ion channels are pharmacologically opened or closed, this can in turn push macrophages towards or away from one inflammatory phenotype. There may also be a correlation between hyperpolarization of peritoneal macrophages and tumor burden in mice (103). As described above, maintenance of resting membrane potential depends on ion channel activity and ion concentrations within and outside of the cell. Elevated potassium ions are observed in murine and human tumor interstitial fluid, with elevated concentrations corresponding to fewer anti-tumor macrophages (104). Macrophages sense elevated potassium in the TME through the ion channel Kir2.1, triggering metabolic reprogramming and an immunosuppressive macrophage phenotype (104).

As reviewed by Barman and Jhunjhunwala, various electrical field stimulation regimens and electrically active materials can be used to alter macrophage phenotype and function (105). This has received particular attention in the context of wound healing, where the induction of an anti-inflammatory, pro-healing phenotype via electrical stimulation can facilitate tissue repair (105), as demonstrated by electrically charged materials mimicking a bone wound (98). Macrophages are also sensitive to applied electric fields, with different waveforms resulting in enhanced activation of cytokine-induced pro- or anti-inflammatory phenotype (106). Therapies to target the tumors by modulation of electrical fields or state should therefore take macrophages into account to maximize anti-tumor efficacy.

T cells have also been shown to migrate cathodally, even in the presence of competing chemotactic cues (48) (107–109). In fact, *ex vivo* observations of a range of peripheral blood lymphocyte subsets revealed consistent cathodal migration (107). The characteristic necrotic core of solid tumors releases potassium ions into the extracellular space, which impairs T cell activation through the T cell receptor and effector function, which can be rescued by increasing potassium efflux (110, 111). Electric fields of physiologically relevant magnitudes can also suppress the activation and proliferation of T cells in response to activating stimuli (108). Similarly, alternating applied electric fields can also suppress T cell function, which may be therapeutically useful in preventing cytokine storm (112). However, electrical-based strategies can also enhance anti-tumor T cell activity. When in contact with a charged surface, T cells and CAR-T cells become more proliferative and have enhanced anti-tumor activity (113).

The response of other immune cell types to physiological electric fields has been less well-studied. Monocytes, macrophage precursors, migrate cathodally (99, 107). Other studies reported that electric fields augment galvanotaxis by neutrophils, though the direction depends on the strength of the applied field (114, 115),. Together, these findings suggest that therapeutic modulation of the electric polarization of both cancer and immune cells may be a strategy to mitigate the immunosuppressive TME. The paucity of work on the effect of endogenous electric fields on neutrophils, dendritic cells, and natural killer cells highlights the need for more research into the significance of the electrical environment on the innate immune system.

## Measurement of electrical properties for cancer diagnosis

There are two main challenges to electrical diagnosis methods for cancer, as outlined by Payne et al. (116). The first is the adaptation of tools designed to study the rapid and robust electrical state changes of neurons and other excitable cells to non-excitable cancer cells, for which the observable electric dynamics are generally slower and subtler (117). The second is the difficulty in understanding the complex relationship between electrical properties and clinically relevant outcomes such as cancer initiation, progression, treatment response, and metastasis. However, technological advances have risen to meet this challenge. In recent years, numerous publications have demonstrated the promise of dielectric characterization, bioimpedance spectroscopy, and microwave tomography as non-invasive and accessible techniques for cancer detection (24). The response of cancer cells and tissues to applied electric fields or currents can reproducibly distinguish them from their normal counterparts, with promise for diagnostic use (24).

Electrical impedance spectroscopy (EIS) measures the impedance, or tendency to oppose electrical current, of a cell or tissue in response to an alternating applied voltage (118). Techniques based on the measurement of electrical impedance have become widely accepted for cancer diagnosis, as they are objective, less invasive, and more time- and cost-effective than standard biopsies (28). Impedance is typically measured using devices with electrodes that are placed in contact with the tissue surface. These electrodes apply alternating current and measure voltage, generating a readout of the resistance of the tissue to the current (119). Tissues respond differently to alternating current; lower frequency currents are thought to flow around the cells and reflect extracellular properties, while higher frequency currents penetrate cell membranes and reflect intracellular properties (120). Not all tumor types have the same change in impedance; for example, prostate tumors that disrupt the otherwise electrically conductive extracellular space cause an increase in measured impedance, while breast tumors have decreased impedance compared to the normally high-impedance, poorly conducting adipose-rich breast tissue (119).

EIS is particularly convenient in assessing tumors that are accessible external to the body, though new methods are under development for its use in endoscopic settings (119, 121). Because of the variability between patients and measurement systems, it is often necessary to compare measurements from the tumor to corresponding healthy tissue from the same patient (119). The use of machine learning algorithms to process EIS data may increase accuracy for cancer in tissues with complex geometry and subtle changes in impedance (119, 121, 122).

In some skin lesion types, EIS has a higher sensitivity and a lower false positive rate in melanoma diagnosis than traditional methods (123, 124). Nevisense, an EIS technology for detecting melanoma, has also been shown to be safe and accurate (125). When used in conjunction with artificial intelligence analysis, EIS can distinguish between normal and malignant oral tissues with high sensitivity and specificity (122). EIS has also shown promise in breast cancer (126), and even low numbers of breast cancer cells can be detected by EIS using a nanoparticle approach (127). This method could be used to detect breast cancer cells in the blood, which is a prognostic marker for breast cancer as well as a metric for its metastatic potential (128). The sensitivity of EIS is comparable to conventional histological analysis in identifying cell density changes in rat intestinal tissue (129). A related technology, electrical impedance tomography (EIT), uses electrical impedance measurements to generate images and has the potential for diagnostic use in several cancer types (130). Its ability to generate high-contrast images of a tumor and amenability to measurements from needle probes placed deep in the tissue give it potential for applications such as image-guided administration of radiation (131).

## Targeting the electrical tumor microenvironment

### Targeting tumors via externally applied fields

While electrophysiological methods in oncology have been the subject of research since the end of the 19th century, early findings lacked key evidence and clinical translation (132). Advances in recent decades in electrically-based therapies have led to patient benefits as seen in clinical trails for glioblastoma (GBM), pancreatic cancer, melanoma, lung cancer, and others (133, 134). Tumor treating fields (TTFs), for example, are alternating current electric fields applied from an external device to target a tumor (134). It is hypothesized that TTFs disrupt the mitotic spindle, alter ion channel activity and increase cancer cell permeability, stimulate autophagy, induce DNA damage, resulting in cell cycle arrest and immunogenic cell death (134). TTF treatment has shown significant benefits in GBM patients, and has since been expanded to include many tumor types (135). The FDA has recently approved a new TTF device, Novocure’s Optune Pax, for pancreatic cancer after a phase III clinical trial showed improved overall survival in combination with chemotherapy (136). Similar technology – Optune Glio and Optune Lua – is approved for glioblastoma, and mesothelioma and non-small cell lung cancer, respectively (137).

In contrast to the low-power, continuous stimulation used for TTFs, pulsed electric fields (PEFs) involve short, high-intensity pulses of electrical stimulation. At sufficiently high doses, this causes irreversible electroporation (IRE), irreversibly damaging cell and organelle membranes and leading to cell death and the activation of the immune system against the resulting damage-induced cell signals (138). This has the potential to overcome major limitations of conventional therapies, such as drug resistance, off-target toxicity, and drug delivery. These strategies may also alter the electrical properties of the target tissue, potentially allowing for responsive monitoring of the ablated tissue region (85). The NanoKnife system, for example, is clinically-approved to induce IRE in tumor settings, and has undergone clinical trials for a range of cancer types with promising results (139). The use of lower-intensity pulses to create temporary pores in the cell membrane, either to facilitate drug entry into the cell, or disrupt ion gradients and trigger apoptotic cell death has proven efficacious (138). The effects on external electrical stimulation on cells, organelles, and downstream signaling pathways is reviewed elsewhere (138). Various electrical stimulation methods are summarized in **Table 1**.

**Table 1:** Modalities of pulsed electric field treatments for solid cancers.

Treatment	Description	Mechanism of action	Pros/cons	Advances
Irreversible Electroporation (IRE)	Electric fields are applied to tissues in 20–200 pulses of 70-100 µs duration, 1000–2000 V/cm strength (140).	Induces nanoscale pores in cell membranes. The electric fields applied are at critical magnitudes such that treated cells are unable to repair their membranes and die, typically by apoptosis (140). At lower voltages, this technique can also be used to create permeations in the cell membrane to allow drug entry (141).	Pros: Effective technique for non-thermal ablation of tumors, some immunogenic effects (141). Clinically approved (139). Cons: Does not discriminate between normal and cancer cells. May cause muscle spasms, bleeding, ulcers, and myocardial infarction, and is less immunogenic than nsPEFs (139, 141, 142).	Image guidance tools allow for precise percutaneous placement of probes for more efficient solid tumor ablation (143). Research is ongoing to optimize the combination with existing chemotherapeutic and immunomodulatory drugs by enhancing delivery in poorly vascularized tumors (144).
High-Frequency Irreversible Electroporation (H-FIRE)	Electric fields are applied in 50–200 bursts containing 25-300 pulses of 1-2 µs duration, 500–4000 V/cm strength (140).	Similar to IRE, but does not cause muscle spasms due to increased frequency. High fields within the cell interior are produced, with effects that scale with nucleus size. Due to nuclear enlargement being associated with malignancy, cancer cells can be targeted with correctly selected frequencies (145).	Pros: Spares more healthy tissue than standard IREs, without causing muscle spasms. Less pain was reported than IRE (141). Cons: Less immunogenic effects than nsPEFs (141).	Effectively increases survival and immune infiltration, especially at higher doses and in combination with chemotherapy, in cells and rodents (146, 147).
Nanosecond Pulsed Electric Fields (nsPEFs)	Electric fields are applied in 10–100 pulses of 10–300 ns duration, 20–150 kV/cm strength (140).	The rise time for pulses is quicker than the time it takes for a cell membrane to recharge (148). The effect of this is that these pulses can penetrate the cell membrane, causing damage to internal structures that leads to apoptosis, without causing excitation of nerve and muscle cells.	Pros: In addition to not causing muscle spasms as much as IRE, nsPEFs are also highly immunogenic (141). Cons: Shorter pulses mean that a stronger field must be generated to observe effects (149).	Mouse xenograft studies have shown nsPEFs can yield a significant reduction in tumor growth rate and modify the immune microenvironment to make it less immunosuppressive (150). Advances in semiconductors and nanomaterials have to potential to increase accessibility (135).
Tumor-Treating Fields (TTFs)	Alternating electric fields at frequencies of 100–300 kHz at an intensity of less than 2 V/cm (151)	May interfere with mitosis through rotationally exciting proteins involved in forming the cleavage furrow (151). Since this method targets cytokinetic cells, it leaves dormant/non-dividing cells undamaged.	Pros: Can aid in chemotherapeutic permeability across the blood-brain barrier, and is non-invasive (152). For example, when treating glioblastoma, it can be applied to a shaven head during everyday activities without a significant change to quality of life. Cons: Modest efficacy, limited by tumor location in body (134). Dangers to patients when combined with radiotherapy due to potential dermatological complications (153).	TTFs impact cancer cell permeability – could enhance drug delivery to cancer cells (154). TTFs can interfere with voltage-gated channels, cause cell cycle arrest, break down mitochondrial membrane potential, and cause DNA degradation and apoptosis in studies with glioblastoma lines (103, 104).

The complex response of the tumor to PEF therapies and their interactions with concurrent therapies must be taken into account for maximum efficacy. For example, the immune response to IRE is generally weak as a monotherapy but combining it with checkpoint blockade leads to complete eradication of primary tumors and metastases in a murine model of lymphoma (117). When IRE is administered at an insufficient dose, it causes the infiltration of immunosuppressive macrophages, leading to tumor recurrence (155). Xu et al. took advantage of this post-treatment macrophage recruitment, creating vesicles that incorporated macrophage membrane proteins and trafficked to the inflamed tumor site to achieve improved delivery of the cargo drugs (155). When the vesicles were loaded with gemcitabine and a CCR2 antagonist and administered after IRE treatment, they induced tumor cell death, inhibited the infiltration of pro-tumor macrophages, and extended survival in murine models of pancreatic cancer (155). In human patients with pancreatic cancer, IRE in combination with allogenic γδ T cell therapy has led to improved overall survival (156).

Determining the safety of electrical stimulation has unique challenges. Charge density, electric field strength, electrode surface area, stimulation waveform, pulse frequency, duty cycle, time requirements, and thickness and conductivity of tissue all play a role in determining safety (105). For example, pulsed electric fields of 50–100 mV/mm are considered safe, as are pulsed current treatments when in the range of 10–50 Hz. Frequencies lower than 1 Hz may stimulate cells for growth, but frequencies in the megahertz range may cause tissue heating and damage (105). Because the efficacy of electrical-based treatments often relies on secondary effects such as immune modulation rather than direct tumor cell killing, treatment regimens may not need to approach safety limits to achieve efficacy (105) (157),. For example, a recent study demonstrated that high-frequency, low strength induced electric fields (1000 times less intense than TTFs) that can be administered without skin contact and with no adverse effects, reprograms the immune TME to reduce primary and metastatic tumor growth (158).

### Electrically active nanomaterials and devices

PEF strategies are inherently limited by limited tissue penetration and damage to surrounding tissue, leading to the exploration of electrically responsive materials for improved efficacy (138). While not yet in clinical use, there is a growing field of research to use properties of nanomaterials to overcome the limitations of externally applied fields (138). Piezoelectric nanomaterials are one such example. Piezoelectricity is a property of materials that accumulate a voltage difference between crystal faces in response to applied mechanical, thermal, or magnetic energy (159). This ability to convert one form of energy to another is useful in the context of medicine, particularly in nanoscale delivery forms that can be injected or implanted and then externally activated. As reviewed elsewhere, piezoelectricity has been exploited for a panoply of medical applications, including drug delivery, imaging, thermal therapy, immunotherapy, tumor-treating fields, and more (160, 161).

A common method of mechanical stimulation of piezoelectric materials is through ultrasonic waves, which are safe, noninvasive, and spatially-targeted (162). Implantable ultrasound stimulators or advanced ultrasound energy-harvesting piezoelectric devices are under development to bypass ultrasound-attenuating structures such as the skull (163, 164). Ultrasound irradiation of biological tissues induces cavitation, causing the deformation of an injected piezoelectric material, which responds by generating a local electrical field (165). Ultrasound activation is advantageous because it is readily available in the clinical setting, unlike larger electric field-generating devices, and can be targeted and localized to the site of interest. The material constituents of piezoelectric materials must be biocompatible and tuned to generate an electric field at frequencies safe for the clinic.

Piezoelectric materials are often used to generate reactive oxygen species (ROS) to eliminate tumor cells (166). Black phosphorous has been recognized as a valuable sonodynamic sensitizer to generate hydrogen peroxide *in situ*. In mouse models bearing 4T1 primary breast tumors, black phosphorous nanosheets injected intratumorally and subjected to ultrasound exposure led to a significant reduction in tumor growth and metastatic potential (166). Recent advances have paired sonodynamic therapy with radiotherapy using hydrogen peroxide-generating nanocatalysts. In another study, polyacrylic acid-bound bismuth oxychloride nanosheets were used to generate hydrogen peroxide *in situ* through ultrasound activation in 4T1 mouse tumor models (167). Hydrogen peroxide generated through this process enhanced the effects of X-ray tumor irradiation, resulting in substantially greater tumor damage than in groups in which either ultrasound-generated hydrogen peroxide or X-ray irradiation was used alone. In another combinatorial approach, He et al. leveraged piezoelectric materials to not only generate cytotoxic ROS, but also to trigger voltage-gated calcium channels in tumor cells, leading to calcium influx and immunogenic cell death (168). This approach bypasses the toxicity concerns of pharmacologically induced calcium overload.

Advanced piezoelectric materials have been developed to be finely tuned for use in the hypoxic tumor environment. For example, Lv et al. report a material that combines bismuth ferrite and bismuth titanate, balancing the proportion of their respective reactions to simultaneously generate oxygen and ROS (169). Upon ultrasound stimulation, the material catalyzes reactions to increase local oxygen tension to alleviate hypoxia and promote reactive oxygen species that damage and kill tumor cells. This resulted in reduced hypoxic signatures in mouse tumors and slowed tumor growth. This material also has favorable properties to serve as a computed tomography and magnetic resonance imaging contrast agent.

The production of ROS that results from piezoelectric activation can be harnessed to initiate other useful reactions at the tumor site. For example, arginine conjugated to strontium-doped barium titanate piezoelectric nanoparticles can be decomposed by ROS to generate nitric oxide (NO), which is cytotoxic and results in improved murine 4T1 tumor control *in vivo* (170). In another combination treatment strategy, a barium titanate nanoparticle was conjugated to both arginine and camptothecin (a chemotherapeutic drug) using a ROS-sensitive bond. Ultrasound activation of the material triggered the production of ROS, releasing camptothecin and catalyzing the production of nitric oxide (NO) by arginine (171). Ultrasound activation of the material caused the production of ROS and NO and improved camptothecan penetration into the tumor, resulting in decreased tumor size in a subcutaneous murine pancreatic cancer model.

Recently, several groups have shown that treatment with piezoelectric materials causes immunogenic cell death and is an effective strategy for immune modulation. Perovskite Ba(Zr_025_Ti_0_._75_)O_3_ nanocubes not only killed tumor cells via ROS generation, but also reprogrammed the immunosuppressive TME by promoting CD8^+^ T cells and anti-tumor macrophage phenotype (172). Separately, piezoelectric nanomotors can penetrate hypoxic solid tumors, where they produce NO and repolarize hypoxic tumor-associated macrophages (173). A hollow-structured piezoelectric material was shown to generate ROS within tumors, causing ferroptosis and decreasing pro-tumor macrophages (174). Piezoelectric materials can also be used to directly reprogram macrophage phenotype, based on whether the particle is intracellular or surface-bound on the target macrophage (175). Finely-tuned combinations of ultrasound stimulus and the resulting electrical stimulus from an ultrasound-activated piezoelectric material can synergize to modulate macrophage phenotype and extracellular vesicle production and content (176).

There is a diverse range of other methods of electrical modulation of the TME in addition to piezoelectrics, including those that leverage distinct material properties and device design, such as triboelectric and pyroelectric materials, electrodes used for electrochemical catalysis, implantable galvanic cells, and magnet-induced eddy current generation (138). For example, Yoshida et al. used microcurrent stimulation to manipulate macrophage circadian rhythm, preventing the natural cyclic reduction in phagocytosis *in vitro* and *in vivo* (177). While still preliminary, this work demonstrates the potential benefits of highly targeted, mechanistically informed electrical immune manipulation. Hu et al. have developed a triboelectric nanomaterial that can deliver pulsed direct current to damage tumor cells via calcium overload (178). This leads to immunogenic cell death, resulting in a more effective anti-tumor T cell response, macrophage recruitment, and dendritic cell maturation and antigen presentation. Similarly, Pan et al. achieved calcium overload and immune activation using a flexible microneedle array for alternating current electrical stimulation (179). The use of “electroactive” materials and recent advances in enabling technology is well-reviewed elsewhere (138).

### Pharmacological targeting of the eTME

“Electroceuticals,” or ion channel modulators, have emerged as repurposed eTME-targeting agents (180). Ion channel modulators are the third best-selling group of prescription drugs worldwide, and there are an estimated 400 ion channels that can be clinically targeted (181). Ligand- and voltage-gated ion channels are one of the most popular targets for current FDA-approved drugs (182). Thus, there is a movement within the field to repurpose ion channel modulators for novel applications in cancer therapy and other bioelectric-driven pathologies.

For example, pantoprazole, a proton pump inhibitor, is used to treat patients with gastric acid hypersecretion due to its ability to irreversibly inhibit the proton pump (H+/K+-ATPase) in active gastric parietal cells (183). It may have potential as an anti-cancer drug in glioma due to its ability to force mitochondrial apoptosis and reduce Nuclear Factor Kappa-B (NF-κB) signaling *in vitro* (184, 185). However, more research is needed to understand its interaction with cancer cells and cancer treatments, as proton pump inhibitors have been associated with poorer patient outcomes in glioblastoma (186, 187). NS1643 is a human-ether-a-go-go-related gene activator with the potential to treat cardiac disorders (188, 189). NS1643 has also been shown to be effective against triple-negative breast cancer metastasis due to its ability to dephosphorylate Caveolin-1 and can be repurposed for glioblastoma treatment (190). Retigabine is a potassium channel drug that is traditionally used for epilepsy treatment, as it reduces neuron excitability (191, 192). Retigabine was withdrawn from the market in 2017 due to reports of side effects, including potential drug-induced liver injury (193, 194) but there are ongoing efforts to redesign its chemical structure to prevent the formation of harmful metabolites *in vivo* (194). Various combinations of these three electroceutical drugs, among others, in human glioblastoma cells demonstrates the potential for synergy between them, with 13 different drug combinations showing superior efficacy compared to temozolomide alone *in vitro* (195). These treatments, when combined optimally, can hyperpolarize cancer cells, arresting proliferation *in vitro.* More broadly, targeting sodium, potassium, calcium, iron, and chloride ion channels increases chemosensitivity across a wide range of cancer types, highlighting promise of electroceutical targeting as part of a combinatorial treatment approach (76).

## Conclusions and outlook

From development to healthy organ function to disease states, bioelectrics play an integral role in cell and tissue function. Electrical dysregulation in cancer has implications for disease progression and, importantly, for diagnosis and treatment. Targeting the eTME has never been more in reach, particularly with the existing FDA-approved drugs that can be repurposed as electroceuticals, in combination with novel technologies. The electrical properties of tumors also hold the promise of providing a non-invasive and accessible means of cancer diagnosis.

However, much remains unknown about the eTME. Some metrics, such as membrane capacitance, yield opposing results, with some studies finding higher capacitance and others finding lower capacitance of cancer cells compared to normal cells. Cytoplasmic conductance changes with malignancy and drug treatment, but whether it increases or decreases with treatment is drug-dependent. The electrical properties of cells are highly microenvironment-dependent, making it difficult to synthesize data from different *in vitro* experiments, particularly in facile but non-physiologic 2D models. These gaps may warrant advanced data analytics techniques and personalized treatment strategies that take into account many variables the clinical setting. A greater mechanistic understanding of the biological significance of measurable electrical properties and their predictive power is needed.

The immune system plays a crucial role in tumor progression or regression, and, like all cell types, immune cells are impacted by endogenous and applied electrical fields. However, key immune response mediators such as dendritic cells and natural killer cells remain understudied in the context of tumor-driven electrical phenomena. As with cancer cell electrical properties, the response of an immune cells to an applied electrical field is often inconsistent between studies, experimental conditions, and the magnitude of the field. It is therefore difficult to establish a clear understanding of the effect of tumor electrics on the immune system. A more systematic and comprehensive effort to characterize the immune system response and role in the eTME would aid in better understanding the anti-tumor immune response and developing improved immunotherapies. This is also true for other organ-specific stromal cell populations that comprise the TME.

While electrical stimulation has demonstrated efficacy in certain settings, it is still hampered by uncertain safety and efficacy characteristics in many cancer types, difficulty in achieving stable, consistent stimulation, and the lack of a clear understanding of its mechanism of action (196). There is a wealth of new research using electroactive nanomaterials to overcome some of these limitations, for example, though they have not yet reached the clinic. The clinical translation of these new approaches faces challenges including the risk of material degradation in the body and after repeated stimulation, the risk of unintended thermal damage to the tissue, and potential activation of a foreign-body reaction by the immune system (197).

Despite these limitations, there have been many recent and exciting advances in eTME basic and translational research. Nanomaterials-based approaches bypass some of the challenges of externally applied fields, with the potential for superior specificity, efficacy, and safety compared to currently approved strategies. Electrical-based therapies have the potential to be highly accessible and widely adoptable, as electrical stimulation is already present in the clinic. The trove of already FDA-approved ion channel-modulating drugs may contain candidates that can be repurposed for eTME reprogramming. Electrical properties can also be measured externally and non-invasively, rendering electrics-based diagnostic methods adaptable to low-resource settings. Patient-specific data analysis powered by artificial intelligence has already enhanced diagnostic accuracy for cancer and could be used to more accurately predict the most effective therapeutic approach. As this is a highly tunable treatment modality, the parameters of electrical treatment could be customized to each patient’s needs as part of a personalized, precision medicine approach. Actualizing this in the clinic will require collaboration between researchers and clinicians with expertise in electrical engineering, materials science, oncology, immunology, and a range of other disciplines. A concerted, multidisciplinary effort from the scientific community will be required to bring these goals to fruition and to elevate the eTME not only to being a well understood cancer hallmark, but also an attractive and vulnerable clinical target.
